# Häufung von Komplikationen der akuten Otitis media und Sinusitis bei Kindern 2022/2023

**DOI:** 10.1007/s00106-023-01393-9

**Published:** 2023-12-18

**Authors:** Noemi Voß, Nadia Sadok, Sarah Goretzki, Christian Dohna-Schwake, Moritz F. Meyer, Stefan Mattheis, Stephan Lang, Kerstin Stähr

**Affiliations:** 1 Klinik für Hals‑, Nasen‑, Ohrenheilkunde, Universitätsklinikum Essen (AöR), Hufelandstraße 55, 45147 Essen, Deutschland; 2 Zentrum für Kinder- und Jugendmedizin, Universitätsklinikum Essen (AöR), Hufelandstraße 55, 45147 Essen, Deutschland

**Keywords:** Akute Mastoiditis, Akute Rhinosinusitis, Orbitale Komplikationen, COVID-19, Jugendliche, Acute mastoiditis, Acute rhinosinusitis, Orbital complications, COVID-19, Teenager

## Abstract

**Hintergrund:**

Akute Mastoiditiden und orbitale Komplikationen der akuten Rhinosinusitis gehören zu den häufigsten Komplikationen pädiatrischer Infektionen in der Hals-Nasen-Ohren-Heilkunde.

**Ziel der Arbeit:**

Ziel dieser Studie war es, die Häufigkeit von akuter Mastoiditis und orbitalen Komplikationen der akuten Rhinosinusitis bei Kindern nach Aufhebung der COVID-19-Sozialdistanzierung im Vergleich zu vor Beginn der Pandemie zu untersuchen.

**Material und Methoden:**

In die Studie eingeschlossen wurden alle Kinder mit akuter Mastoiditis und orbitalen Komplikationen bei akuter Sinusitis im Zeitraum von April 2017 bis März 2023, die am untersuchten Universitätsklinikum stationär behandelt wurden. Die drei Zeiträume von April 2017 bis März 2020 (vor der Pandemie in Deutschland), April 2020 bis März 2022 (während der Kontaktbeschränkungen in der Pandemie) und April 2022 bis März 2023 (nach Aufhebung der Kontaktbeschränkungen) wurden mittels deskriptiver Statistik miteinander verglichen.

**Ergebnisse:**

Insgesamt wurden 102 Kinder (43 mit akuter Mastoiditis, 42 %, und 59 mit orbitalen Komplikationen einer akuten Sinusitis, 58 %) eingeschlossen. Im Zeitraum 2022/2023 wurden mehr als doppelt so viele Kinder mit akuter Mastoiditis und circa dreimal so viele Kinder mit orbitalen Komplikationen einer akuten Rhinosinusitis stationär behandelt im Vergleich zum Durchschnitt der Zeiträume 2017/2018, 2018/2019 und 2019/2020. Im Zeitraum 2020/2021 lag die Anzahl dieser Patient:innen unter dem Durchschnitt der vorangegangenen Jahre.

**Schlussfolgerung:**

Die diesjährige saisonale Häufung von Infekten der oberen Atemwege geht einher mit einem überdurchschnittlichen Auftreten von orbitalen Komplikationen und Mastoiditiden.

## Abstract (en)

**Background:**

Acute mastoiditis and orbital complications of acute rhinosinusitis are among the most common complications of pediatric infections in otolaryngology.

**Objective:**

The aim of this study was to investigate the frequency of pediatric acute mastoiditis in the setting of acute otitis media as well as pediatric orbital complications in the setting of acute rhinosinusitis. Data from before the pandemic were compared to data after the end of the COVID-19 restrictions.

**Materials and methods:**

Included were hospitalized children who presented with acute mastoiditis from acute otitis media or with orbital complications from acute rhinosinusitis during the period from April 2017 to March 2023. Compared were three periods using descriptive statistics: April 2017 to March 2020 (before the pandemic in Germany), April 2020 to March 2022 (during the contact restrictions of the pandemic), and April 2022 to March 2023 (after the contact restrictions were lifted).

**Results:**

A total of 102 children (43 with acute mastoiditis, 42%, and 59 with orbital complications of acute sinusitis, 58%) were included. During the 2022/2023 period, more than twice as many children with acute mastoiditis and approximately three times as many children with orbital complications of acute rhinosinusitis were hospitalized compared to the average of the periods 2017/2018, 2018/2019, and 2019/2020. In the 2021/2022 period, the number of these patients was below the average of previous years.

**Conclusion:**

This year’s seasonal cluster of upper respiratory tract infections is associated with a higher-than-average incidence of orbital complications and mastoiditis.

Die COVID-19-Pandemie hat die Infektionslage weltweit und damit auch in Deutschland grundlegend verändert. Zu Beginn führten Kontaktbeschränkung und Maskenpflicht zu einem deutlichen Rückgang von Atemwegsinfekten bei Kindern mit aktuellem deutlichem Anstieg nach Wegfall der Beschränkungen im Alltag. Auch in der Hals-Nasen-Ohren-Heilkunde konnte im Winter 2022/2023 ein deutlicher Anstieg akuter Mastoiditiden und orbitalen Komplikationen bei akuter Rhinosinusitis bei Kindern beobachtet werden, was in dieser Studie untersucht wird.

Zu Beginn der COVID-19-Pandemie war ein signifikanter Rückgang oberer Atemwegsinfektionen bei Kindern durch die Kontaktbeschränkungen, Maskenpflicht und allgemeine Hygienemaßnahmen zu verzeichnen [3, 11]. Dies hat sich auch in der Hals-Nasen-Ohren-Heilkunde auf die Inzidenz von Infektionen wie die akute Otitis media und akute Rhinosinusitis ausgewirkt, die ebenfalls signifikant zurückgingen [10, 19].

In den Jahren davor hatte sich die Inzidenz der akuten Mastoiditis und akuten Rhinosinusitis bei Kindern weitestgehend stabil gezeigt. Im Winter 2022/2023 zeigt sich eine hohe Rate an Atemwegsinfekten bei Kindern, die in den Kinderkliniken zu einer außergewöhnlichen Steigerung der Hospitalisierung und damit Belastung der Kliniken führte [3].

Die akute Mastoiditis ist die häufigste Komplikation der akuten Otitis media und tritt vor allem im Kindesalter auf. Die Inzidenz liegt bei 1–4 pro 100.000 Einwohner/Jahr. Zugrunde liegt fast immer eine akute Otitis media. Mehr als zwei Drittel aller Kinder entwickeln bis zum dritten Lebensjahr mindestens eine Episode einer akuten Otitis media. Die akute Otitis media zählt damit zu den häufigsten Infektionen im Kindesalter [20]. Das Risiko für weitere Komplikationen wie Meningitis, intrakranielle Abszesse oder Sinusvenenthrombose, mit möglicherweise lebensbedrohlichen Folgen, ist dabei nicht zu unterschätzen. Daher ist das schnelle Einleiten einer gezielten Therapie essenziell [8].

Die Therapie der akuten Mastoiditis besteht immer in einer stationären Behandlung und intravenösen Antibiotikagabe. Operativ ist bei intaktem Trommelfell mindestens eine Parazentese mit Paukendrainage, Epipharyngoskopie und ggf. Adenotomie durchzuführen. Bei ausgedehntem Befund erfolgt zusätzlich eine Mastoidektomie [13].

Orbitale Komplikationen sind die häufigste Form von Komplikation der akuten Rhinosinusitis und treten ebenfalls vor allem im Kindesalter auf [21]. Dabei erfolgt die Therapie stadienabhängig rein konservativ oder operativ mit begleitender intravenöser Antibiotikagabe und abschwellenden Nasentropfen [15].

An dem untersuchten Universitätsklinikum wurde im Winter 2022/2023 eine deutliche Zunahme der komplikativ verlaufenden Sinusitiden und Otitiden im Kindesalter beobachtet. Daher ist das Ziel dieser Studie, die Zunahme der Häufigkeit von akuter Mastoiditis und orbitalen Komplikationen bei akuter Rhinosinusitis bei Kindern nach Wegfall der Kontaktbeschränkung und Maskenpflicht im Alltag durch COVID-19 zu untersuchen.

## Material und Methoden

### Studiendesign, Teilnehmer und Prozedere

In diese retrospektive Studie wurden alle Kleinkinder, Kinder und Jugendliche (< 18 Jahre) mit orbitalen Komplikation bei akuter Rhinosinusitis sowie akuter Mastoiditis bei akuter Otitis media, die in der Periode von April 2017 bis März 2023 im untersuchten Universitätsklinikum stationär behandelt wurden, inkludiert. Andere Komplikationen auf dem Boden einer akuten Rhinosinusitis oder akuten Otitis media wurden ausgeschlossen.

Das Studienprotokoll entspricht der Erklärung von Helsinki und wurde von der örtlichen Ethikkommission genehmigt (Nummer: 22-11045-BO).

Es erfolgte eine zentrale Abfrage der Patientendaten nach entsprechender internationaler statistischer Klassifikation der Krankheiten und verwandter Gesundheitsprobleme (ICD-10; Codes: H70.0, H05.0, J01.0–J01.9) beziehungsweise Operationen- und Prozedurenschlüssel (OPS-Code; Codes: 5‑203.0, 5‑22, 5‑160). Ausgeschlossen wurden Patient:innen mit inkompletten Datensätzen.

### Endpunkte und Definitionen

Der primäre Endpunkt dieser Studie war die Untersuchung der Häufigkeit von akuten Mastoiditiden und orbitalen Komplikationen bei akuter Rhinosinusitis in den letzten sechs Jahren. Der sekundäre Endpunkt war die Veränderung des durchschnittlichen Alters der Kinder in den einzelnen Zeiträumen.

Des Weiteren wurden das Alter (in Jahren), Geschlecht (männlich oder weiblich), Therapieart (konservativ und oder operativ), Verweildauer (in Tagen), Seite (links oder rechts oder ohne Angabe), Grad der Komplikation (im Fall einer orbitalen Komplikation bei akuter Rhinosinusitis) sowie das ermittelte Keimspektrum ausgewertet.

Die orbitalen Komplikationen bei akuter Rhinosinusitis wurden anhand der Klassifikation von Chandler in 5 Schweregrade eingeteilt. Stadium eins beschreibt dabei eine präseptale Zellulitis, Stadium zwei eine orbitale Zellulitis, Stadium drei einen subperiostalen Abszess, Stadium vier einen orbitalen Abszess und Stadium fünf eine entzündlich bedingte Sinus-cavernosus-Thrombose [4].

### Analyse

Für die Auswertung der Daten wurden Gruppen von April bis einschließlich März der entsprechenden Jahre gebildet. Dabei wurden Daten von April 2017 bis einschließlich März 2023 eingeschlossen. Hierbei sollte die jeweils in den Wintermonaten stattfindende Häufung von Grippe- und sonstigen Rhinosinusitiden innerhalb einer Gruppe erhoben werden.

Es wurden die drei Zeiträume von April 2017 bis März 2020 (vor der COVID-19-Pandemie in Deutschland), April 2020 bis März 2022 (während der Kontaktbeschränkungen in der COVID-19-Pandemie in Deutschland) und April 2022 bis März 2023 (nach Wegfall der Kontaktbeschränkungen in der COVID-19-Pandemie in Deutschland) miteinander verglichen.

Bei geringer Gruppengröße wurde auf die Berechnung von statistischen Signifikanzen verzichtet und lediglich deskriptive Statistik verwendet, um die Ergebnisse zu beschreiben. Zur Einordung der Häufigkeiten wurde der Median sowie der Interquartilsabstand (IQR) ermittelt.

## Ergebnisse

In die Studie konnten insgesamt 102 Kinder (43 Kinder mit akuter Mastoiditis, 42 %, 59 Kinder mit orbitalen Komplikationen bei akuter Sinusitis, 58 %) eingeschlossen werden. Das Alter lag bei akuter Mastoiditis im Median bei 3 Jahren und bei orbitalen Komplikationen bei 7 Jahren. Von den Kindern waren 62 männlich (61 %) und 40 weiblich (39 %). Nur bei insgesamt 4 der 43 Kinder (9,3 %) lag eine beidseitige akute Otitis media vor, bei den anderen 39 Kindern (90,7 %) eine einseitige akute Otitis media. Alle Kinder wurden entweder in der Klinik für Hals‑, Nasen‑, Ohrenheilkunde oder der Klinik für Kinderheilkunde stationär behandelt (Tab. 1).

**Table 1:** 

	Zeitraum^a^					
	2017/2018	2018/2019	2019/2020	2020/2021	2021/2022	2022/2023
*Anzahl der Patient:innen*						
Akute Mastoiditis	4	8	8	–	5	18
Akute Otitis media einseitig/beidseitig	4/0	8/0	8/0	–	4/1	15/3
Orbitale Komplikationen	5	6	10	2	10	26
*Alter im Jahre* *(Median (Spannweite))*						
Akute Mastoiditis	5,5 (3–15)	3,5 (1–9)	3 (0–6)	–	2 (1–9)	3 (0–14)
Orbitale Komplikation	7 (1–12)	6 (3–13)	7,5 (0–17)	5,5 (3–8)	8,5 (2–16)	8 (0–17)
*Geschlecht (männlich* *=* *m, weiblich* *=* *w)*						
Akute Mastoiditis	4 m, 0 w	5 m, 3 w	4 m, 4 w	–	4 m, 1 w	9 m, 9 w
Orbitale Komplikation	4 m, 1 w	4 m, 2 w	7 m, 3 w	2 m, 0 w	5 m, 5 w	14 m, 12 w
*Betroffene Seite (rechts* *=* *r, links* *=* *l, ohne Angabe* *=* *o.A.)*						
Akute Mastoiditis	1 r, 3 l	2 r, 6 l	3 r, 5 l	–	2 r, 3 l	6 r, 12 l
Orbitale Komplikation	3 r, 2 l	2 r, 4 l	5 r, 5 l	0 r, 2 l	7 r, 2 l, 1 o.A.	12 r, 14 l
*Verweildauer (Tage; Median (Spannweite))*						
Akute Mastoiditis	5,5 (3–10)	4,5 (2–17)	5,5 (3–17)	–	9 (7–16)	5 (2–31)
Orbitale Komplikation	4 (3–7)	4 (3–5)	5 (2–9)	6,5 (5–8)	4 (2–7)	4 (1–30)

– Im Zeitraum 4 (2020/2021) stellte sich kein Patient:in mit akuter Mastoiditis am untersuchten Universitätsklinikum vor

^*a*^Zeiträumen jeweils April bis März des Folgejahrs

### Akute Mastoiditis

Bei einer akuten Mastoiditis erfolgte bei insgesamt 4 Kindern eine rein konservative Therapie mit intravenöser Antibiotikagabe und abschwellenden Nasentropfen, alle anderen Kinder erhielten eine zusätzliche operative Therapie. Diese bestand bis auf wenige Ausnahmen in einer Mastoidektomie mit Parazentese und Paukendrainage der betroffenen Seite. Dabei erhielten alle Patient:innen < 16 Jahre zusätzlich zumindest eine Nasenracheninspektion und bei Vorliegen von Adenoiden eine zusätzliche Adenotomie.

**Figure 1:**
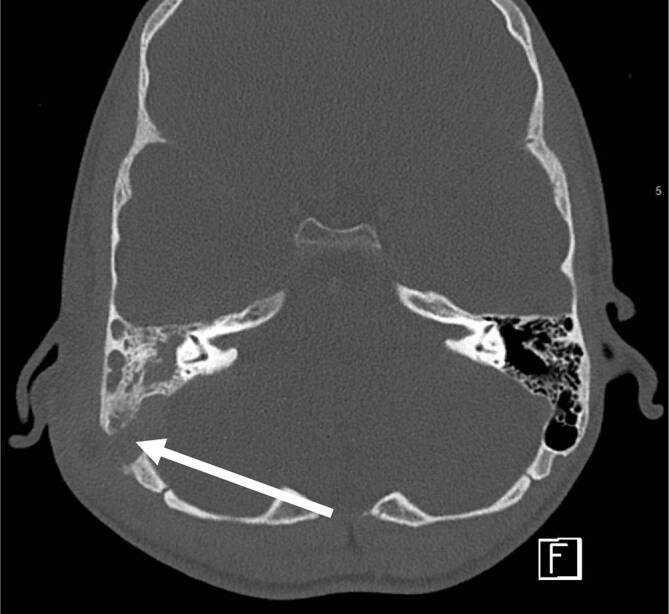

In der Computertomographie zeigt sich dabei eine Verlegung und knöcherne Destruktion im Bereich der Mastoidzellen (Abb. 1).

**Figure 2:**
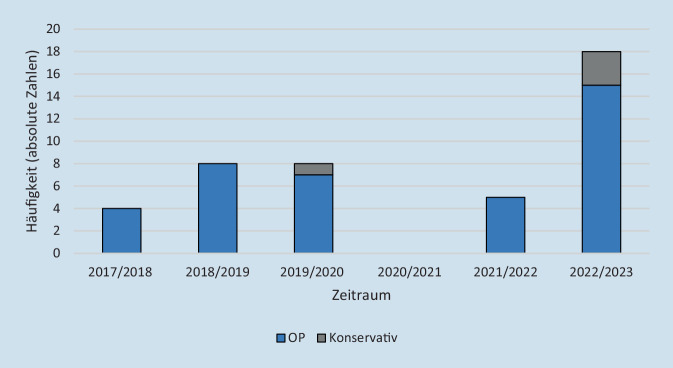

Im Zeitraum von April 2017 bis März 2020 zeigten sich im Median 8 Fälle (IQR: 2) pro zwölfmonatigen Zeitraum. Im ersten Pandemiezeitraum von April 2020 bis März 2022 stellten sich im Median 2,5 Kinder (IQR: 2,5) pro zwölfmonatigen Zeitraum mit akuter Mastoiditis vor. Im Zeitraum von April 2022 bis März 2023 stellten sich 18 Kinder mit akuter Mastoiditis vor. Damit stellten sich im letzten untersuchten Zeitraum mehr als doppelt so viele Kinder mit akuter Mastoiditis im Vergleich zu vor Beginn der COVID-19-Pandemie in Deutschland vor (Abb. 2).

Im mikrobiologischen Abstrich konnte bei 10 von 12 Kindern (89 %) mit akuter Mastoiditis seit Beginn Dezember 2022 und erfolgter zusätzlicher operativer Therapie entweder Streptococcus pyogenes (6 Kinder) oder Streptococcus pneumoniae (4 Kinder) nachgewiesen werden. Davor war entweder kein erfolgreicher Keimnachweis möglich, oder es zeigten sich Streptococcus pneumoniae, Streptococcus pyogenes und Pseudomonas aeruginosa sowie in einzelnen Fällen andere Bakterien. Dabei war kein eindeutiger Trend zu einem der genannten Erreger sichtbar.

### Orbitale Komplikation

**Figure 3:**
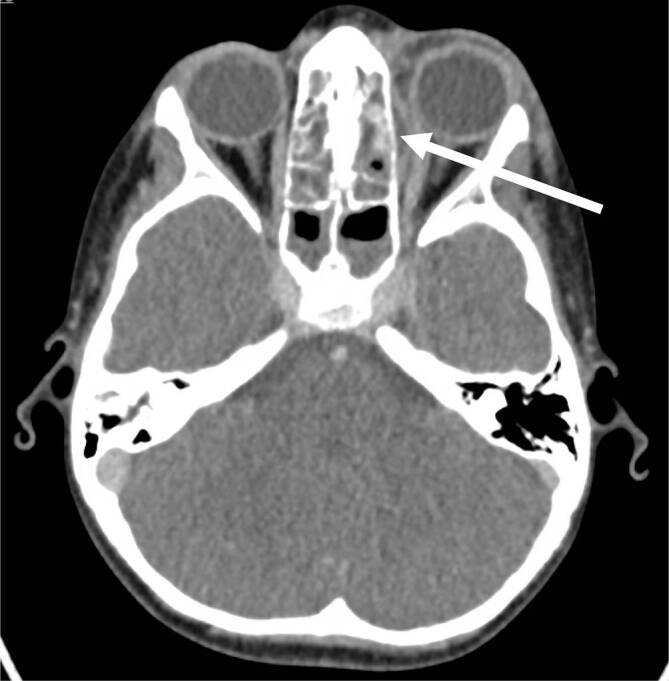

Bei Kindern mit orbitaler Komplikation bei einer akuten Rhinosinusitis erfolgte die Therapie stadienabhängig. Insgesamt zeigte sich im Zeitraum nach der Pandemie (Zeitraum von April 2022 bis März 2023) ein Anstieg der Häufigkeit orbitaler Komplikationen in Stadium I und III. Die genaue Häufigkeit der jeweiligen Stadien kann aus Tab. 2 entnommen werden (Tab. 2). In der Computertomographie zeigt sich im Stadium III dabei eine Verschattung der Nasennebenhöhlen mit angrenzendem Verhalt subperiostal im Bereich der Orbita (Abb. 3). Dabei erfolgte in Stadium I und II bei 28 von 30 Kindern (93 %) eine konservative Therapie mit intravenöser Antibiose und abschwellenden Nasentropfen. Bei allen Kindern ab Stadium III erfolgte eine zusätzliche operative Therapie. Diese bestand aus einer endoskopischen Nasennebenhöhlenoperation mit Abszessentlastung im Bereich der Orbita. Bei 8 von 30 (27 %) operativ behandelten Kindern war eine zusätzliche Abszessentlastung von außen über einen Augenbrauenrandschnitt bzw. einer Inzision lateral des lateralen Kanthus erforderlich.

**Table 2:** 

	Zeitraum^a^					
	2017/2018	2018/2019	2019/2020	2020/2021	2021/2022	2022/2023
Anzahl der Kinder mit orbitaler Komplikation	5	6	10	2	10	26
Stadium der orbitalen Komplikation nach Chandler (I–V)	I: 3 II: 0 III: 2 IV: 0 V: 0	I: 5 II: 0 III: 1 IV: 0 V: 0	I: 4 II: 1 III: 5 IV: 0 V: 0	I: 1 II: 0 III: 1 IV: 0 V: 0	I: 6 II: 0 III: 4 IV: 0 V: 0	I: 11 II: 0 III: 14 IV: 1 V: 0

^a^Zeiträume jeweils April bis März des Folgejahres

**Figure 4:**
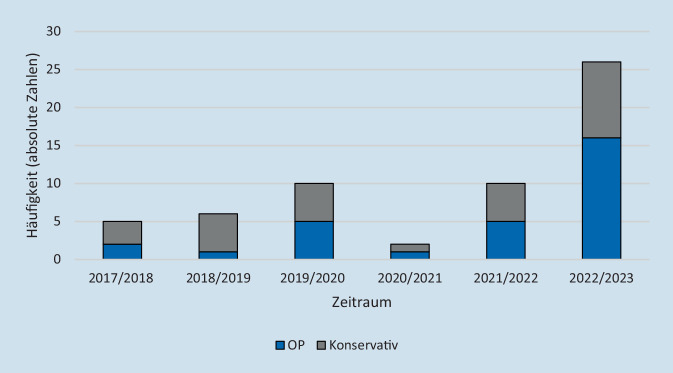

Im Zeitraum von April 2017 bis März 2020 zeigten sich im Median 6 Fälle (IQR 2,5) pro zwölfmonatigem Zeitraum. Im ersten Pandemiezeitraum von April 2020 bis März 2022 wurden im Median 6 Fälle (IQR 4,5) pro zwölfmonatigem Zeitraum behandelt. Im Zeitraum von April 2022 bis März 2023 wurden 26 Kinder mit orbitaler Komplikation bei akuter Rhinosinusitis behandelt. Damit war im letzten untersuchten Zeitraum die Anzahl der Patient:innen mehr als dreimal so hoch wie vor Beginn der COVID-19-Pandemie (Abb. 4).

Im mikrobiologischen Abstrich konnte in allen Jahren vor allem Staphylococcus aureus und Streptococcus pyogenes sowie Streptococcus intermedius ohne eindeutige Dominanz eines bestimmten Erregers nachgewiesen werden.

## Diskussion

Diese monozentrische Studie zeigt erstmalig einen deutlichen Anstieg der Häufigkeit von akuten Mastoiditiden und orbitaler Komplikationen bei akuter Rhinosinusitis bei Kindern in der winterlichen Krankheitswelle 2022/2023. Zudem konnte eine deutliche Reduktion dieser Komplikationen im Zeitraum von April 2020 bis März 2021 beobachtet werden. In den Jahren vor Beginn der COVID-19-Pandemie waren die Inzidenzen akuter Mastoiditiden und orbitaler Komplikationen bei akuter Rhinosinusitis bei Kindern weitestgehend stabil [2, 7].

Der Rückgang der akuten Rhinosinusitis und akuten Otitis media entspricht dem in anderen Untersuchungen beobachteten Rückgang an kindlichen Atemwegsinfektionen vor allem in den Jahren 2020/2021 [1, 10, 19]. Dies wird vor allem auf die durch die COVID-19 bedingten Kontaktbeschränkungen, Maskenpflicht und allgemeine Hygienemaßnahmen zurückgeführt [9, 23].

Der Anstieg der orbitalen Komplikationen und Mastoiditiden im Winter 2022/2023 entspricht der derzeit diskutierten überdurchschnittlichen Rate an Infektionen der oberen Atemwege [3, 18]. Eine ausführliche wissenschaftliche Aufarbeitung steht ist bislang aus.

Neben der Pädiatrie war auch in unserer Klinik für Hals‑, Nasen‑, Ohrenheilkunde eine deutliche Zunahme von stationären Behandlungen im Kindesalter in der winterlichen Krankheitswelle 2022/2023 zu verzeichnen. Methi et al. prognostizierten eine derartige Entwicklung und begründeten diese Voraussage mit einer zu erwartenden fehlenden Exposition zu typischen Krankheitserregern bedingt durch Hygienemaßnahmen [16]. Während der noch reduzierten Infektionszahlen bei Kindern in der COVID-19-Pandemie haben Studien die Möglichkeit eines Rebound-Effekts nach Wegfall der Kontaktbeschränkungen vorausgesagt [1, 5, 22]. Es ist denkbar, dass dieser Effekt derzeit in Deutschland zu beobachten ist.

In unserer Studie lag das durchschnittliche Alter bei Kindern mit akuter Mastoiditis im Zeitraum April 2020 bis März 2023 bei 3,4 Jahren. Betroffen waren also vor allem Kinder, die kurz vor Beginn der COVID-19-Pandemie geboren wurden und somit durch die Kontaktbeschränkungen, Maskenpflicht und allgemeine Hygienemaßnahmen zunächst möglicherweise eine geringe Exposition zu Keimen hatten. Vor der COVID-19-Pandemie (April 2017 bis März 2020) lag das durchschnittliche Alter in unserer Untersuchung bei 4,3 Jahren. Dies könnte durch die fehlende Ausbildung der erworbenen Immunantwort durch die verringerte Keimexposition in der COVID-19-Pandemie zu einer nun erhöhten Rate an kompliziert verlaufenen Infektionen bei kleinen Kindern geführt haben [5]. Eine derartige Verminderung des durchschnittlichen Alters bei orbitalen Komplikationen bei akuter Rhinosinusitis ließ sich allerdings nicht verzeichnen. Es zeigte sich in unserer Untersuchung ein durchschnittliches Alter von 8,1 Jahren im Zeitraum April 2020 bis März 2023. Das mittlere Alter der Kinder mit orbitaler Komplikation bei einer akuten Rhinosinusitis lag vor Beginn der COVID-19-Pandemie in unserer Untersuchung bei durchschnittlich bei 7,4 Jahren, was in etwa dem durchschnittlichen Alter bei Kindern mit orbitaler Komplikation bei einer akuten Rhinosinusitis vor Beginn der COVID-19-Pandemie entspricht [14, 17].

Streptococcus pyogenes oder Streptococcus pneumoniae konnte seit Dezember 2022 bei fast allen Kindern mit akuter Mastoiditis in dieser Studie nachgewiesen werden. Bei den orbitalen Komplikationen auf dem Boden einer akuten Rhinosinusitis bei Kindern konnten in dieser Studie Staphylococcus aureus, Streptococcus pyogenes und Streptococcus intermedius am häufigsten nachgewiesen werden. Damit entsprechen die nachgewiesenen Keime den häufigsten Erregern der beiden Erkrankungen [6, 12]. Der Anstieg der Häufigkeit der beiden Krankheitsbilder lässt sich somit nicht durch außergewöhnliche Erreger erklären und stützt die Vermutung, dass das erworbene Immunsystem durch die Kontaktbeschränkungen und Maskenpflicht nicht ausreichend trainiert wurde [5].

Die Limitation dieser Studie ist zum einen, dass es sich um eine monozentrische Untersuchung handelt, sodass theoretisch auch eine Verschiebung der Fallzahlen von umliegenden Kliniken zu der beschriebenen Erhöhung der Häufigkeiten geführt haben könnte. Zum anderen wurden insgesamt über einen Zeitraum von 6 Jahren 102 Kinder eingeschlossen, sodass auf die Berechnung von statistischen Signifikanzen bei geringer Gruppengröße verzichtet wurde.

## Fazit für die Praxis

Eine deutliche Erhöhung der Häufigkeit von akuter Otitis media und akuter Rhinosinusitis sowie deren Komplikationen bei Kindern in der winterlichen Infektionswelle 2022/2023 führte zu einer Mehrbelastung der HNO-Kliniken.
